# The adoption paradox: Exploring the role of ethnicity, deprivation, and co-ethnic density in care proceedings in England

**DOI:** 10.23889/ijpds.v8i2.2252

**Published:** 2023-09-14

**Authors:** Sam Lindsay, Ross Kennedy, Tom Hepworth, Andy Bond

**Affiliations:** 1 Ministry of Justice, London, United Kingdom

The study objectives were to (1) examine the association between children’s ethnicity and final legal orders at the end of family care proceedings (section 31 of the 1989 Children Act), and (2) test whether residential context, such as co-ethnic density and area-level deprivation, moderates this association.

Two sources of data were used for this study. The first consisted of records routinely generated by Cafcass (England) and stored in the Secure Anonymised Information Linkage (SAIL) databank, and the second was the 2021 England Census. The focus was on children whose initial care proceedings took place between 2015/2016 and 2020/2021 and concluded with a recorded final legal order outcome (N = 98,161). Three-level logistic regression models were employed to estimate the relationship between children's ethnicity and adoption, along with the potential moderating effects of co-ethnic density and area-level deprivation.

Children's ethnicity is significantly associated with placement for adoption, with white children being more likely to be subject to placement orders compared to children from all other ethnic groups combined (Asian, black, mixed or multiple, and other ethnic groups). Higher local authority co-ethnic density considerably reduces the likelihood of adoption for children of other ethnicities besides white, but not for white children. Moreover, white children living in the most deprived LSOAs are more likely to be placed for adoption than those residing in the least deprived LSOAs. However, the likelihood of placement for adoption remains consistent across all LSOA deprivation quintiles for children from ethnicities other than white. Local authority-level deprivation does not appear to moderate the relationship between children's ethnicity and adoption.

This study sheds light on the intricate relationship between ethnicity, residential context, and adoption. While previous research has indicated that white children are more likely to be adopted, the findings enhance our understanding of the underlying mechanisms influencing adoption, paving the way for a more equitable family justice system.

